# Establishing Embryogenic Tissue Culture Workflow for Pineapple Cultivar 73–50

**DOI:** 10.3390/genes16050549

**Published:** 2025-04-30

**Authors:** Ming Cheng, Yuri Trusov, Guoquan Liu, Yanfei Mao, Jose Ramon Botella

**Affiliations:** 1 Plant Genetic Engineering Laboratory, School of Agriculture and Food Sustainability, The University of Queensland, St Lucia 4072, Australia; ming.cheng@uq.edu.au (M.C.); y.trusov@uq.edu.au (Y.T.); 2 Centre for Crop Science, Queensland Alliance for Agriculture and Food Innovation, The University of Queensland, St Lucia 4072, Australia; g.liu2@uq.edu.au; 3 CAS Center for Excellence in Molecular Plant Sciences, Chinese Academy of Sciences, Shanghai 200031, China; yfmao@sibs.ac.cn

**Keywords:** pineapple tissue culture, pineapple transformation, pineapple embryogenic callus

## Abstract

**Background**: The development of an efficient tissue culture system is essential for advancing genetic transformation and genome editing in commercially important pineapple cultivars. However, a robust tissue culture workflow for the elite pineapple cultivar 73–50, enabling reliable transformation and plant regeneration is not established. **Methods**: A comparative analysis of hormone combinations, including 6-benzylaminopurine (BAP), α-naphthaleneacetic acid (NAA), picloram, and abscisic acid (ABA) was conducted. Transformation competence of 73–50 callus was tested using the *iGUS* reporter gene. **Results**: We established that 1 mg/L picloram and 0.5 µg/L ABA was the most effective combination for inducing friable embryogenic callus (FEC). FEC, composed of small, loosely associated cell clusters, is highly suitable for transformation but prone to browning during long-term culture. We optimized the conditions to minimize browning and support prolonged maintenance using a medium supplemented with 5 mg/L NAA. Transformation efficiency was demonstrated using the *iGUS* reporter gene, showing that FEC can be effectively transformed via both biolistic and *Agrobacterium*-mediated methods. For shoot regeneration, the optimal medium was found to contain 2 mg/L BAP. To standardize the assessment of callus development, we introduce a classification system describing distinct developmental stages. **Conclusions**: A detailed step-by-step protocol optimized for 73–50 cultivar facilitates efficient genetic improvement in pineapple, supporting both conventional transformation and DNA-free genome editing approaches.

## 1. Introduction

Pineapple (*Ananas comosus* L. Merrill), a tropical species commonly known for its fruit, has significant economic, nutritional, and cultural value. Native to South America, pineapple has become a global commodity, with major production in Southeast Asia, Central America, and Africa [1]. In 2022, over 80 countries contributed to pineapple cultivation, resulting in an estimated annual production of 29.36 million tons, as reported by the Food and Agriculture Organization of the United Nations (FAOSTAT). Over the last two decades, global pineapple output has increased by 217.5%, while the cultivation area has expanded by 177.5% [2]. A major factor in this growth has been the development of sweet pineapple varieties such as MD1 (commonly known in Australia as 73–50) and MD2 (also labeled as 73–114), whose appealing taste has driven consumer demand. These cultivars are characterized by high yield, low acidity, and enhanced resistance to internal browning [3,4]. However, further improvements are needed to reduce premature flowering, extend shelf life, and enhance disease resistance. These hybrid cultivars originate from a complex breeding program involving Smooth Cayenne, Smooth Guatemalan, Ruby (a clone of Singapore Spanish), and Queen varieties [5]. Additionally, the 73–50 contains about 13% ‘Pernambuco’ ancestry [6]. Despite extensive tissue culture studies on many of its parental cultivars, 73–50 still lacks fully developed, reliable, and effective tissue culture protocols.

Pineapple propagation is essential for ensuring the high-quality and large-scale production of planting material. Most commercial pineapple varieties reproduce exclusively through vegetative propagation, using suckers, slips, and crowns. However, their low multiplication rate poses a significant challenge for the industry [7,8]. Tissue culture including micropropagation using axillary buds [9] and multiplication from callus provides an alternative method for rapid clonal propagation, allowing for the large-scale production of genetically uniform plants. In addition to its role in propagation, tissue culture is a critical tool for genetic improvement through transformation or CRISPR/Cas9-mediated genome editing [10].

A key requirement for crops’ genetic improvement is the development of a robust embryogenic callus culture system. Embryogenic callus serves as a suitable material for transformation and DNA-free genome editing techniques, due to its high capacity for taking up transforming agents and its ability to produce genetically uniform plantlets. While friable embryogenic callus has been successfully produced in the Smooth Cayenne cultivar [11], the tissue culture characteristics of 73–50 remain unexplored. A major challenge in establishing an effective system is optimizing the medium conditions to promote callus induction while minimizing browning and maintaining the callus in a proliferative state suitable for transformation.

In this work, we aimed to develop an efficient tissue culture system for the pineapple cultivar 73–50 to support genetic transformation and DNA-free genome editing approaches. Specifically, our objectives were to establish conditions for the induction and maintenance of embryogenic callus, along with regeneration protocols, and create a classification system to standardize the assessment of callus developmental stages. To address these aims, we developed a detailed tissue culture workflow tailored to cultivar 73–50. Using the *iGUS* reporter gene, we demonstrated that embryogenic callus can be successfully transformed via either biolistic or Agrobacterium-mediated methods. Altogether, our findings provide a foundational system to facilitate future genetic studies and crop improvement efforts in pineapple.

## 2. Materials and Methods

### 2.1. Plant Material and Callus Induction

Plants of the pineapple cultivar 73–50 were provided by Tropical Pines Pty. Ltd., Hidden Valley, QLD, Australia, and collected from fields in South Queensland, Beerwah, Australia (-26.883189244773487, 152.92237906697494) during June–August 2023. Crowns from mature plants were transported to the laboratory and kept on the bench in dry trays at ambient temperature (approximately 25 °C) for up to 14 days until processing.

Pineapple crowns were stripped of their leaves, and the crown cores were washed under running tap water for 20 min to remove dirt, debris, and other contaminants. Axillary buds and the white bases of the inner leaves were excised and surface-sterilized by washing twice with 0.5% sodium hypochlorite and 0.05% Tween 20 for 10 min. Sterilized buds and leaf bases were rinsed five times with sterile water and placed on solid callus induction medium (CIM) in plastic Petri dishes (90 mm diameter × 15 mm height) containing approximately 50 mL of medium. The CIM consisted of 4.4 g/L Murashige and Skoog (MS) modified basal medium with Gamborg vitamins (M404, Phytotech Labs, Lenexa, KS, USA), 0.1 g/L myo-inositol, 36.7 mg/L FeNaEDTA, 30 g/L sucrose, and 2.75 g/L Phytagel (Sigma, Ronkonkoma, NY, USA), supplemented with 10 mg/L each of 6-benzylaminopurine (BAP) and α-naphthaleneacetic acid (NAA), adjusted to pH 5.7.

Plates with tissue were kept in the dark at 25 °C for one week. Subsequently, the tissue was maintained under cool-white fluorescent lamps (approximately 30 µmol/m^2^/s) with a 16/8 h photoperiod at 25 °C. Rough globular callus appeared within approximately one month and was maintained on CIM for at least two to three months, with monthly sub-culture before further experiments.

### 2.2. Media for Embryogenic Callus Development

To improve the efficiency of both DNA-free and transformation-mediated genome editing, tissue cultures should contain small cell clusters (5–100 µm in diameter) with the potential for somatic embryo formation. This developmental stage, previously described as embryogenic cell clusters [11], is crucial for successful regeneration. To promote the transition of rough callus to an embryogenic state in the 73–50 cultivar, we tested several hormone combinations, as listed in Table 1. The base medium for all treatments consisted of 4.44 g/L Murashige and Skoog (MS) basal salts, 30 g/L sucrose, 36.7 mg/L FeNaEDTA, 100 mg/L myo-inositol, and 2.75 g/L Phytagel, adjusted to pH 5.7–5.8. To designate specific hormone combinations, we used the first letter of each hormone: B for BAP, N for NAA, P for picloram, and A for ABA. The accompanying number indicates the hormone concentration in mg/L for B, N, and P, or in µg/L for A. For example, 10B10N indicates a medium containing 10 mg/L BAP and 10 mg/L NAA.

### 2.3. Callus Growth Assessment and Statistical Analysis

Each hormonal combination was tested using 10 replicate plates. For each plate, 15 callus masses of approximately 0.5 g were distributed onto solid medium in standard Petri dishes (90 mm in diameter and 15 mm in height). The callus weight was recorded again after 30 days of growth on the respective hormonal medium. Weight gain was calculated as the difference between the initial and final weight for each callus. The number of browned calli was counted per plate, and the progression to the embryogenic stage was assessed prior to weighing. Statistical analyses, including normality and ANOVA tests, were performed using GraphPad Prism (Version 9.3.1, GraphPad Software, Boston, MA, USA) to determine the mean differences among treatments.

### 2.4. Biolistic DNA Delivery

Biolistic DNA delivery was performed as previously described in [13]. Briefly, friable embryogenic callus was spread using forceps at the center of a plate containing osmotic medium (4.44 g/L MS, 0.4 M mannitol, and 15 g/L agar, pH 5.7). The plasmid pCAMBIA1305.1, carrying an intron-containing β-glucuronidase (*iGUS*) gene (Canberra, Australia), was purified using a MaxiPrep kit (QIAGEN, Ann Arbor, MI, USA), following the manufacturer’s instructions, and diluted to a final concentration of 1 µg/µL. A 10 µL aliquot of plasmid DNA was coated onto 0.6 µm gold particles using 2.5 M CaCl_2_ and 0.1 M spermidine, and then washed with 100% ethanol. A 15 µL suspension of coated particles was pipetted onto a microcarrier and dried for 5 min. Bombardment was carried out in a vacuum chamber, with the target tissue positioned 15 cm from the barrel. Helium gas was applied at 1000 kPa with a 0.5-millisecond pulse to accelerate the DNA-coated particles into the plant cells. Friable embryogenic callus was incubated on osmotic medium (4.44 g/L MS, 5 mg/L NAA, 0.4 M mannitol and 15 g/L agar) for at least 3 h prior to bombardment and 18 h after.

### 2.5. Agrobacterium-Mediated Transformation

*Agrobacterium tumefaciens* strain GV3101, carrying the pCAMBIA1305.1 vector, was grown overnight at 28 °C in 5 mL of LB medium supplemented with 50 mg/L rifampicin and 50 mg/L kanamycin. The culture was then diluted to an OD_600_ of 0.1 in 100 mL of LB medium (without antibiotics) containing 100 mM acetosyringone and grown for approximately five hours until reaching an OD_600_ of 0.8. Bacterial cells were collected by centrifugation, washed twice with sterile water, and resuspended in sterile water supplemented with 100 µM acetosyringone to an OD_600_ of 0.5. Friable embryogenic callus was mixed with the Agrobacterium suspension for 20 min on a shaking platform (50 RPM). The callus was then blotted on filter paper and transferred to 1P5A medium for co-cultivation. Co-cultivation was conducted in the dark at 26 °C for 48 h.

### 2.6. Histochemical GUS Staining Assay

The histochemical β-glucuronidase (GUS) assay was performed as previously described in [15]. Briefly, plant tissue (leaves or callus) was collected 5–7 days after bombardment and incubated in GUS staining solution containing 100 mM sodium phosphate buffer (pH 7.0), 0.5 mM potassium ferrocyanide, 0.5 mM potassium ferricyanide, 0.1% Triton X-100, and 0.2 mg/mL X-Gluc (5-bromo-4-chloro-3-indolyl-β-D-glucuronic acid) at 37 °C for 24–48 h. Following incubation, the stained tissues were washed several times with 70% ethanol to remove excess stain and clarify the tissue. The samples were stored in fresh 70% ethanol until further analysis.

## 3. Results and Discussion

### 3.1. Optimization of Hormone Composition for 73–50 Pineapple Callus Maintenance

**Figure 1:**
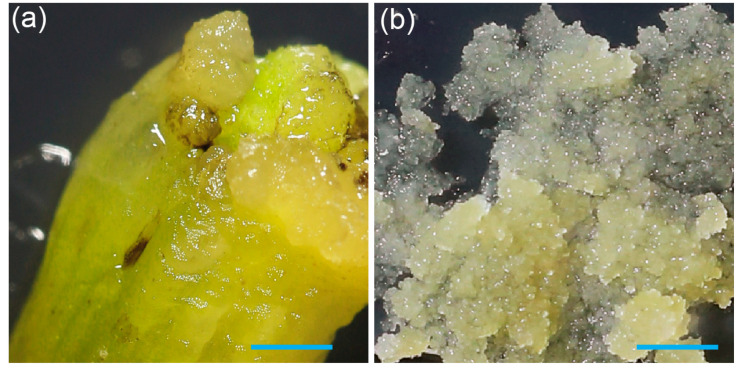
Induction of pineapple callus from leaf base, and formation of friable embryogenic cell clusters: (**a**) Callus formation on the edge of a white leaf base after eight weeks of culture on 10B10N medium. (**b**) Development of friable embryogenic callus on 1P5A medium. The scale bar on both images is 1 mm.

Auxins and cytokinins are key plant hormones that can induce the dedifferentiation of specialized cells, restoring them to a totipotent state. Analysis of existing pineapple tissue culture protocols has shown that different cultivars respond variably to auxin-to-cytokinin ratios [12,14,16,17,18,19,20]. Therefore, optimizing all critical steps, including callus induction, maintenance, and plant regeneration for a specific cultivar, is critical for efficient tissue culture. Using the callus induction protocol from [13]—specifically, the BAP/NAA composition 10B10N—we successfully induced callus formation from 73–50 pineapple white leaf bases (Figure 1a).

Efficient delivery of genetic material or ribonucleoproteins (RNPs) into target cells is a prerequisite for successful transformation and genome editing. Maximizing the target tissue surface area would improve the transformation efficiency and RNP uptake. This can be achieved by generating small embryogenic cell clusters (ECCs), which provide a larger surface area for more uniform exposure to DNA or RNPs, actively divide to enhance the regeneration potential, and minimize the risk of chimeric plants and somaclonal variation. Production of ECCs has been previously reported for Smooth Cayenne pineapples using picloram and abscisic acid [11,21]. To generate embryogenic cell clusters (ECCs) in the 73–50 cultivar, two-month-old callus initially obtained on 10B10N (Figure 1a) was transferred to a medium supplemented with 1P5A (see Section 2 Materials and Methods for medium details). After approximately 30 days, we observed the formation of friable ECCs (Figure 1b).

**Figure 2:**
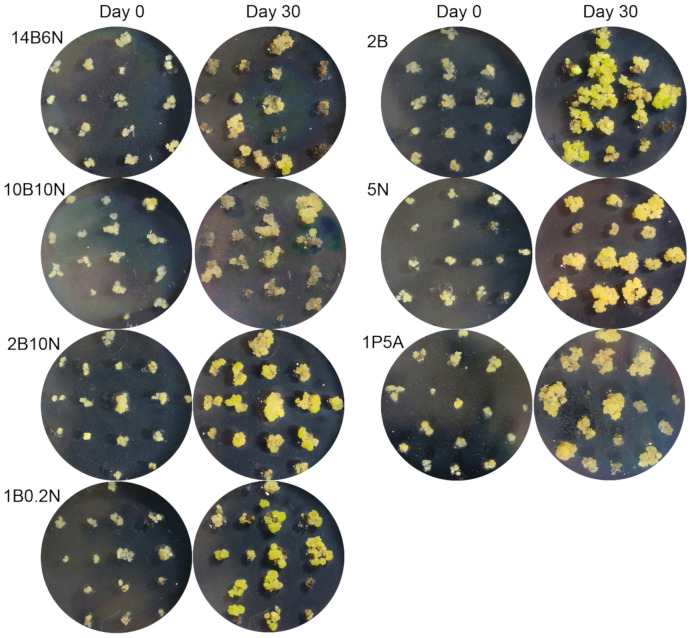
Embryogenic callus growth and development on seven media. Callus was transferred to media with different hormonal compositions, as listed in Table 1. One representative plate out of ten replicates was photographed at the time of transfer, and again after 30 days of culture, to illustrate callus development over time.

Once small, friable ECCs were successfully induced, the next challenge was to determine the optimal conditions for their long-term maintenance. We tested seven different hormone compositions (Table 1) to identify which medium best supported ECCs’ proliferation while preserving their structure. Equal amounts of ECCs, split into 15 portions of approximately 0.5 g, were placed on 10 replicate plates for each tested medium (left side photos in Figure 2). After 30 days, the callus was obviously increased in mass (right side photos in Figure 2).

**Figure 3:**
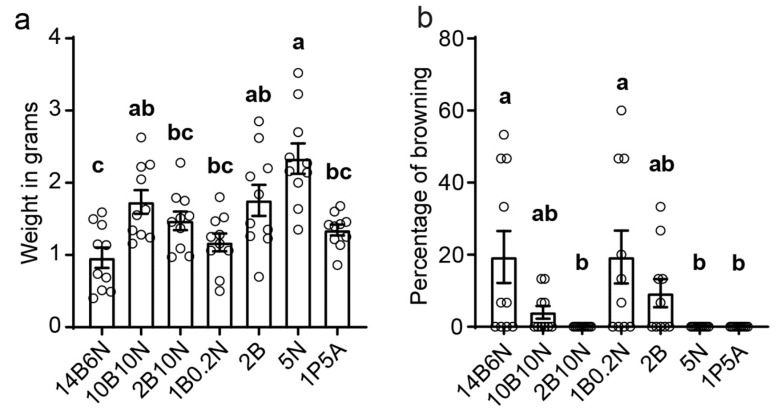
Quantification of embryogenic callus growth and browning on seven media: (**a**) Equal amounts (0.5 g) of FEC were distributed onto plates with the designated hormone combinations. After 30 days, callus was scraped from each of ten replicate plates and weighed. Bars represent the mean ± SEM, *n* = 10. (**b**) The percentage of browned callus was quantified for each plate, and the mean ± SEM of ten replicates is shown. Statistical differences were evaluated using one-way ANOVA followed by Tukey’s test for multiple comparisons. Different letters indicate significantly different groups (*p* < 0.05).

To evaluate the growth, the callus was scraped from each plate and weighed. Among the tested media, 5N yielded the highest callus growth, with a maximum of 3.52 g and an average of 2.34 ± 0.2 g for 10 plates. The next best performers were 10B10N (1.74 ± 0.15 g) and 2B (1.76 ± 0.2 g), followed by 2B10N (1.47 ± 0.12 g), 1B0.2N (1.17 ± 0.12 g), and 14B6N (0.96 ± 0.14 g) (Figure 3a). These results indicate that the 5N medium is the most effective for maintaining friable ECCs.

Callus browning is a common problem in tissue culture, leading to reduced tissue quality, inhibited growth, and eventual tissue death. Throughout our experiments, we occasionally observed callus browning. To quantify the browning in relation to hormone composition, we recorded the percentage of browned callus per plate. The results indicated that media with higher BAP concentrations were more prone to browning (Figure 3b), with 14B6N causing approximately 19% of callus tissues to turn brown. In contrast, no browning was observed in media lacking BAP, such as 5N and 1P5A. The absence of browning in 5N medium, combined with the highest mass gain, establishes it as the optimal choice for maintaining 73–50 pineapple ECCs.

### 3.2. Classification of Developmental Stages of Pineapple Callus

The developmental stage of callus is important for DNA or RNP uptake efficiency, shoot regeneration, and other tissue culture applications. To evaluate callus progression toward plant regeneration, we required simple and easily recognizable stages of callus development. However, to the best of our knowledge, such a classification covering pineapple’s regeneration from callus is not currently available, although a detailed description of embryogenic cell clusters and tissues in pineapple, outlining key morphological criteria, has been reported [11]. Building on this work, we adapted the early-stage classification and introduced additional post-embryogenic stages to provide clearer nomenclature for describing progressive callus development.

**Figure 4:**
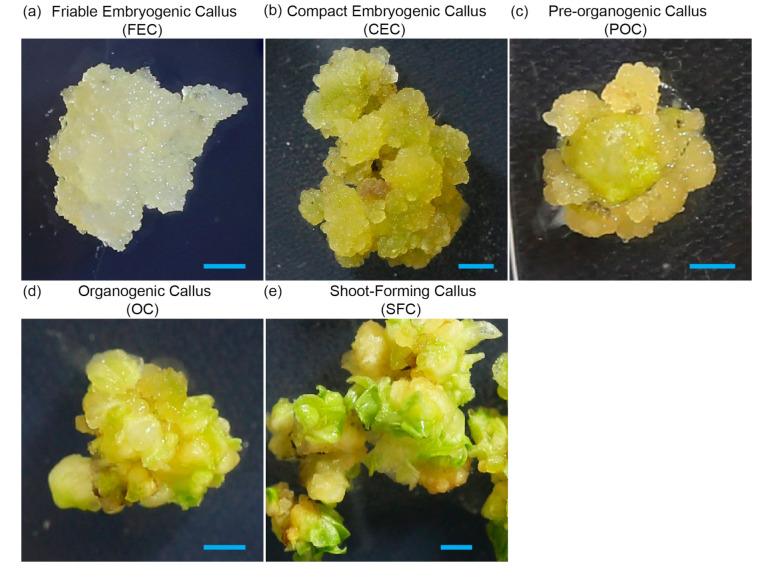
Developmental stages of 35–70 pineapple callus, from cell clusters to shoot formation: (**a**) Friable embryogenic callus (FEC) consists of embryogenic cell clusters. (**b**) Compact embryogenic callus (CEC) consists of embryogenic callus bodies of 3–6 mm. (**c**) Pre-organogenic callus (POC), where smooth and nodular surfaces are visible. (**d**) Organogenic callus (OC) with formation of shoot primordia, where small leaf-like structures are visible. (**e**) Shoot-forming callus (SFC) with small rootless plantlets. Bars = 1 cm.

Our callus development classification consists of five distinct consecutive stages (Figure 4), each reflecting a gradual transition from early embryogenic callus to shoot-forming structures:

1. Friable embryogenic callus (FEC), consisting of loosely aggregated embryogenic cell clusters (20–500 cells, 5–100 µm in diameter). Highly dispersible, without visible structural organization, it is ideal for biolistic or *Agrobacterium*-mediated transformation and RNP uptake. This stage is equal to the ECC described by [11].
2. Compact embryogenic callus (CEC) consists of non-dispersible embryogenic callus bodies of 3–6 mm. It is still suitable for transformation and corresponds to embryogenic tissue in [11].
3. Pre-organogenic callus (POC) consists of more compact structures with the appearance of smooth or nodular surfaces, representing the beginning of cellular organization with cells exhibiting polarity.
4. Organogenic callus (OC) is characterized by formation of shoot primordia, indicating the transition from embryogenesis, with visible leaf-like structures. There are some remaining embryogenic regions, but it is predominantly organogenic.
5. Shoot-forming callus (SFC) is characterized by small rootless plantlets present among leaf-like structures. This is no longer true callus, transitioning to full shoot formation, and is ready for elongation and rooting.

**Figure 5:**
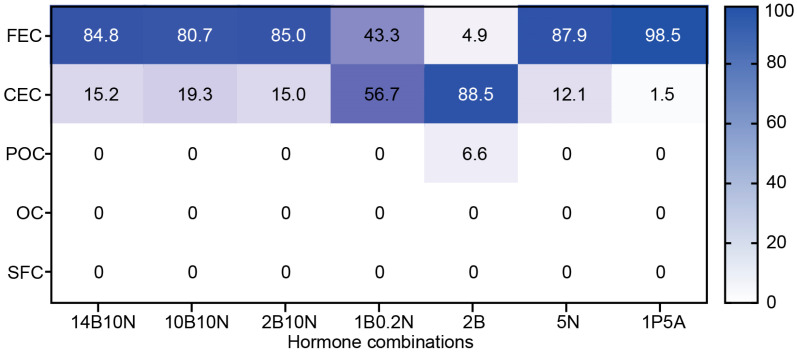
Heatmap showing the distribution of callus across five developmental stages. Callus was cultured on the designated media for 30 days, and the number of calli reaching each developmental stage was recorded. The values represent the average percentage per ten replicate plates.

Using this classification, which aligns with previous studies and provides a practical framework for describing callus developmental stages, we evaluated the developmental progression of 73–50 callus over 30 days on the same seven media. As previously mentioned, equal amounts of FEC were plated on 10 plates per hormone combination and analyzed after 30 days of growth. The heatmap in Figure 5 illustrates the progression of callus development, expressed as the percentage of calli that reached each designated stage on a given medium after 30 days of culture. Among the tested media, 2B was the most effective in promoting callus development, with 88.5% of calli reaching the CEC stage, 6.6% progressing to the pre-organogenic stage, and only 4.9% remaining as the initial FEC. In contrast, no significant developmental progression was observed on 1P5A, where 98.5% of the calli remained at the friable embryogenic stage, represented by ECCs. Media with high NAA concentrations, such as 14B10N, 10B10N, 2B10N, and 5N, allowed approximately 15–20% of calli to reach the CEC stage, while 80–85% remained as the initial FEC. Notably, on 1B0.2N, which contained five times more BAP than NAA, callus development was intermediate between that observed on 2B and the high-NAA media, with 56.7% of calli progressing to the CEC stage.

### 3.3. Establishing Transient Expression in Friable Embryogenic Callus Using Biolistic and Agrobacterium-Mediated Approaches

**Figure 6:**
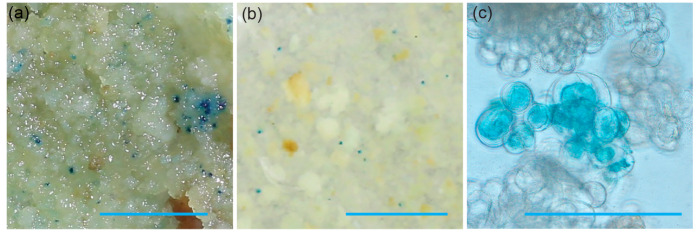
Transient *iGUS* expression assays in friable embryogenic callus after (**a**) biolistic and (**b**) *Agrobacterium*-mediated transformation using the pCAMBIA1305.1 plasmid. The scale bar on both (**a**) and (**b**) images is 5 mm. Micrograph (**c**) shows a single cluster of friable embryogenic callus expressing *iGUS* three days after co-cultivation with *Agrobacterium*. The scale bar is 20 µm.

To demonstrate the ability of the 73–50 friable embryogenic callus to be transformed, we performed a test biolistic and *Agrobacterium*-mediated transformation. The vector pCAMBIA1305.1 with the *β-glucuronidase* (*GUS*) gene containing an intron under the control of the cauliflower mosaic virus 35S promoter [22] was used for both transformation methods. The results of the transient expression of *iGUS* are shown in Figure 6.

### 3.4. Optimized Tissue Culture Procedure for Pineapple Cultivar 73–50

**Figure 7:**
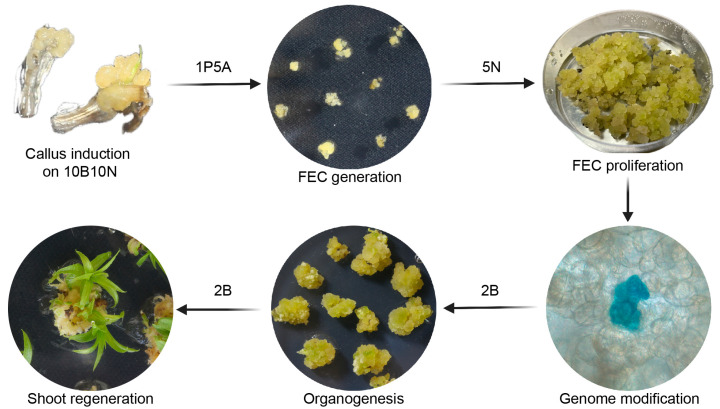
Tissue culture workflow for the 73–50 cultivar. Dedifferentiated callus is induced from the white leaf base on 10B10N medium. Friable embryogenic callus suitable for genetic manipulations is generated on 1P5A medium. FEC can be maintained and proliferated on 5N medium. At this stage, FEC can be used for transformation of DNA-free editing manipulations. Organogenesis and shoot regeneration are achieved on 2B medium.

Based on our data presented above, we suggest the following optimized procedure for handling the pineapple cultivar 73–50: The initial step involves inducing callus from suitable explants, such as white leaf bases or dormant buds. Since white leaf bases are more abundant and have shown comparable callus formation in our hands, we recommend using them over buds. Explants must be sterilized before culturing on 10B10N medium. It is advisable to incubate the explants in the dark for one week before transferring the plates under luminescent lights. After four weeks of incubation, compact callus masses should develop at the cut surfaces of the explants. Once callus is initiated, it should be removed from the leaf tissue and sub-cultured on 1P5A medium to facilitate the transition to embryogenic stages. Embryogenic cell clusters will appear in approximately four weeks, presenting as dense, small aggregates of cytoplasm-rich cells. At this stage, the callus develops into friable embryogenic callus (FEC), characterized by its loose, easily dispersible structure. This friability is crucial for efficient transformation and downstream applications, including genetic transformation and genome editing. Due to its high regenerative capacity and accessibility, FEC can be subjected to biolistic or Agrobacterium-mediated transformation, as well as ribonucleoprotein (RNP)-based genome editing. Following transformation or RNP treatment, the callus can be transferred to selective or non-selective media for recovery and analysis. Successfully transformed or treated callus can be induced to undergo somatic embryogenesis on 2B medium. Within four weeks, approximately 88% of the embryonic tissue should develop into compact embryogenic callus (CEC). Following sub-culture on the same 2B medium, an additional 4–6 weeks are needed for CEC to transition into a mixture of pre-organogenic callus (POC) and organogenic callus (OC). Once small, rootless plantlets appear among the OC, they can be transferred to hormone-free rooting medium until complete plantlets develop. The emergence of roots indicates that the plantlets are ready for transfer to soil for further growth. This workflow is graphically presented in Figure 7. It provides an efficient and reproducible process for generating embryogenic callus, enabling high-throughput transformation and genome editing, followed by successful plant regeneration.

## 4. Conclusions

This study establishes an efficient tissue culture system for the 73–50 pineapple cultivar, supporting future genetic transformation and genome editing applications. We optimized the conditions for embryogenic callus induction and maintenance, demonstrating that the tissue can be successfully targeted by both biolistic and Agrobacterium-mediated delivery. The proposed classification of callus developmental stages provides a practical and standardized framework for assessing callus progression toward regeneration. The complete process, from explant preparation to plantlet regeneration, typically takes approximately eight months and is highly reproducible. Phenotypic evaluation of regenerated plants requires a second generation for reliable comparison with the mother plants, and this aspect is currently a work in progress. Potential limitations of the method include the usual issues for tissue culture plants: somaclonal variation among first-generation regenerants, and the relatively high labor and costs associated with long-term tissue culture. Nonetheless, the established protocol offers a robust platform for advancing genetic improvement in pineapple, facilitating trait enhancement, and accelerating breeding efforts.

## Figures and Tables

**Table 1:** Hormone combinations for embryogenic callus development optimized for 73–50 pineapples.

Name	Hormone Combination	Reference
14B6N	14 mg/L BAP and 6 mg/L NAA	[12]
10B10N	10 mg/L BAP and 10 mg/L NAA	[13]
2B10N	2 mg/L BAP and 10 mg/L NAA	
1B0.2N	1 mg/L BAP and 0.2 mg/L NAA	[14]
2B	2 mg/L BAP	
5N	5 mg/L NAA	
1P5A	1 mg/L picloram and 5 µg/L ABA with 1 g/L glutamine	[11]

## Data Availability

The raw data supporting the conclusions of this article will be made available by the authors upon request.

## Abbreviations

The following abbreviations are used in this manuscript:

LB	Luria–Bertani medium
BAP	6-Benzylaminopurine
NAA	a-Naphthaleneacetic acid
ABA	Abscisic acid
GUS	β-Glucuronidase
iGUS	GUS with intron
RNPs	Ribonucleoproteins
ECCs	Embryogenic cell clusters
CRISPR	Clustered Regularly Interspaced Short Palindromic Repeats
CIM	Callus induction medium
FEC	Friable embryogenic callus
CEC	Compact embryogenic callus
POC	Pre-organogenic callus
OC	Organogenic callus
SFC	Shoot-forming callus
